# Cellular Trace Element Changes in Type 1 Diabetes Patients

**DOI:** 10.4274/jcrpe.2449

**Published:** 2016-06-06

**Authors:** Vahap Uğurlu, Çiğdem Binay, Enver Şimşek, Cengiz Bal

**Affiliations:** 1 Osmangazi University Faculty of Medicine, Department of Pediatrics, Division of Pediatric Endocrinology, Eskişehir, Turkey; 2 Osmangazi University Faculty of Medicine, Department of Medical Statistics, Eskişehir, Turkey

**Keywords:** type 1 diabetes mellitus, trace elements, magnesium tolerance test

## Abstract

**Objective:**

Type 1 diabetes mellitus (T1DM) may lead to deficiencies in trace elements that have substantial functions in the human organism. Changes in serum magnesium (Mg), copper (Cu), and zinc (Zn) levels are correlated with metabolic control and diabetes complications. The aim of this study was to evaluate the intra-erythrocyte levels of trace elements and urinary Mg excretion following intravenous (iv) Mg tolerance testing in children with T1DM.

**Methods:**

A total of 43 children aged 2-18 years with T1DM and age/gender-matched 25 healthy children were included in the study. The iv Mg tolerance test was performed following the measurement of intra-erythrocyte Mg (eMg1), Cu (eCu1), and Zn (eZn1) levels using the atomic absorption spectrophotometer method. The Mg retention ratio was estimated from measurements in 24 h urine samples.

**Results:**

No statistically significant difference was found for eMg1, eCu1, and eZn1 levels between the patient and control groups (p>0.05). In the patient group, the eMg1, eCu1, and eZn1 levels measured after the iv Mg tolerance test significantly increased compared with the baseline levels (p<0.05), and the Mg excretion ratio measured from the urine collected after the iv MgSO4 infusion was >50%.

**Conclusion:**

The increased retention value following the iv Mg tolerance testing indicates intracellular Mg deficiency in children with T1DM.

**WHAT IS ALREADY KNOWN ON THIS TOPIC?**

Magnesium (Mg) is the most commonly seen trace element deficiency in patients with type 1 diabetes mellitus (T1DM). Moreover, Mg deficiency is involved in the pathogenesis of diabetes complications that inhibit the prostacyclin receptor function and cause increased thrombocyte activation and aggregation.

**WHAT THIS STUDY ADDS?**

Intra-erythrocyte Mg (eMg1) measurement cannot reveal Mg deficiency, but increased retention following intravenous Mg tolerance test indicates intracellular Mg deficiency in patients with T1DM.

## INTRODUCTION

Type 1 diabetes mellitus (T1DM) is a chronic metabolic disease that occurs with increasing frequency in children. Recently, the effects of trace elements on glucose metabolism have been reported, suggesting their role in the etiopathogenesis and complications of diabetes (1,2,3).

In many studies conducted on diabetic patients and experimental animals, zinc (Zn) has been reported to have positive effects on hyperglycemia by increasing phosphoinoside-3 kinase activation, serine/threonine kinase phosphorylation, GLUT4 (glucose transporter type 4) translocation and insulin sensitivity and to enhance prevention of development of complications due to diabetes by decreasing oxidative stress (4,5,6,7). Magnesium (Mg) is the most commonly seen trace element deficiency in patients with T1DM. Decreased plasma and tissue Mg levels have been reported among diabetic patients (1,2,8). Mg plays an important role in whole reactions, including cellular energy transfer, glycolysis and phosphorylation, and it prevents free radical generation required to ensure increased glutathione syntheses (1). Moreover, Mg deficiency is involved in the pathogenesis of diabetes complications that inhibit the prostacyclin receptor function and cause increased thrombocyte activation and aggregation (9,10). Body mineral values are ideally determined by measuring their tissue levels.

In the present study, we aimed to evaluate the tissue trace element levels by measuring the intra-erythrocyte Mg (eMg1), Zn (eZn1), and copper (eCu1) levels and the urinary Mg excretion level following the intravenous (iv) Mg tolerance test in children and adolescents diagnosed with T1DM in our pediatric endocrinology department.

## METHODS

Forty-three children and adolescents diagnosed with T1DM between June 2012 and March 2013 in the Department of Pediatric Endocrinology at Eskişehir Osmangazi University Faculty of Medicine (group 1) were included in the study. The inclusion criteria were as follows: (1) age under 18 years, (2) diagnosis of T1DM at least six months prior to admission, and (3) no concomitant disease apart from T1DM. The study also included 25 healthy children and adolescents without any chronic disease as a control group (group 2).

The study protocol was approved by the Ethics Committee of Osmangazi University Faculty of Medicine. Informed consent was obtained from all included children and their parents. A detailed physical examination was performed both in the study and control groups. Systolic/diastolic blood pressure (BP) was measured, and cases with a BP ≥95th percentile were considered hypertensive (11). Body weight (BW) and height were measured in all cases. Height was measured by length gauge scale (Harpenden, Holtain, Crymych, UK), and BW was measured by portable scale (SECA 762; Vogel&Halke, Hamburg, Germany). Body weight and height percentiles were estimated using the age- and gender-appropriate growth curves for Turkish children (12). Body mass index (BMI) was calculated as weight in kilograms divided by the square of length. Cases with ≥95th percentile of BM were considered obese (13).

In both the study and control groups, following a 12-hour fasting period, baseline venous blood specimens were taken for determination of fasting blood glucose, urea nitrogen (BUN), creatinine, calcium (Ca), phosphorus (P), alkaline phosphatase (ALP), total cholesterol (TC), triglyceride (TG), low-density lipoprotein-cholesterol (LDL-C), high-density lipoprotein-cholesterol (HDL-C), and serum Mg levels. Blood specimens were also taken into 2 mL tubes containing EDTA for measurement of eMg1, eZn1, and copper (eCu1) levels.

Hemoglobin A1c (HbA1c) levels were measured in all cases in the study group. HbA1c levels of 6.06%-7.4%, 7.5%-9.0%, and >9.0% indicated satisfactory, mediocre, and poor control, respectively (14). Among the group 1 cases, retinal examination was performed by an ophthalmologist.

In group 1, the 24 h urine specimens were collected into plastic tubes that did not contain any metal for determination of microalbumin, creatinine, and urinary Mg (uMg1) levels. In the control group, urinary Mg level was estimated in spot urine samples.

Following the 24 h urine collection, the patient group was administered 0.2 mEq/kg of elemental Mg. This was given as a 4 h iv infusion of a 15% magnesium sulfate (MgSO4) in a 5% dextrose solution. For ethical reasons, iv MgSO4 was not given to the control group. In the patient group, for measurements of volume, creatinine, microalbumin, and uMg2 levels, 24 h urine specimens were again collected after starting the MgSO4 infusion. At the end of the second 24 h urine collection, eMg2, eZn2, and eCu2 levels were measured again. At the end of the iv MgSO4 infusion, Mg retention was estimated as follows (15):

Mg retention (%) = 1− A- (BxC) / D = x100

A: Mg level in 24 h urine after iv MgSO4

B: Mg/creatinine ratio in 24 h urine before iv MgSO4

C: Urinary creatinine level after iv MgSO4

D: Amount of total elemental Mg infusion

A Mg retention level of >50% indicated deficiency. A possible Mg deficiency was considered when the Mg retention level was 25%-50%. A normal Mg level was considered when the retention level was <25%.

### Laboratory Analysis

eMg1, eCu1, eZn1, and urinary Mg levels were measured using the atomic absorption spectrophotometer (Analyst 100 Flame, Perkin-Elmer). Glucose, Ca, P, ALP, BUN, TG, HDL-C, LDL-C, and Mg levels were estimated using the enzymatic calorimetrical method with Roche Modular Equipment. Serum and 24 h urinary creatinine levels were measured using the kinetic colorimetric method, HbA1c was measured using the turbidimetric inhibition method, and the microalbumin level was measured using the immune turbidimetric method in 24 h urine.

### Statistical Analysis

Data were analysed using the Statistical Package for the Social Sciences 15.0 software for Windows (SPSS Inc., Chicago, IL, USA). The variable distribution was specified using the Kolmogorov-Smirnov test. The parametrical variables were expressed as mean ± standard deviation, and the non-parametrical variables were expressed as median (minimum-maximum). For intergroup comparison, the normally distributed variables were compared using independent samples t-test, and the non-normally distributed variables were compared using the Mann-Whitney U-test. For intragroup comparison, paired samples t-test and Wilcoxon rank test were performed, respectively. The inter-variable associations were determined by the Pearson and Spearman correlations. The intergroup comparison for the qualitative parameters was performed using the chi-square test. The cut-off value was estimated using the receiver operating characteristic (ROC) curve analysis. p<0.05 was considered statistically significant.

## RESULTS

Among the T1DM patients (group 1), 19 (44.2%) were girls and 24 were boys (55.8%). Mean age was 12.8±3.2 years. In the control group (group 2), 17 (68%) were girls and 8 were boys (32%). Mean age was 12.4±4.5 years. Table 1 shows the demographic and anthropometrical characteristics of the two groups. The patient group received insulin lispro three times daily and insulin glargine once a day, and the daily total insulin dose was 0.7 U/kg/day-1.5 U/kg/day. Of these patients, according to HbA1c levels, 4 (9.3%) had well-controlled diabetes, 20 (46.5%) had mediocre-controlled diabetes, and 19 (44.2%) had poorly controlled diabetes.

**Figure 1:**
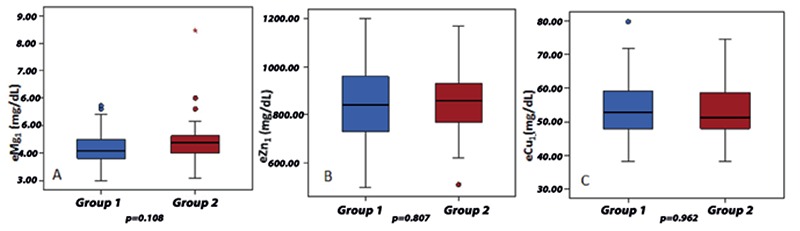
Intergroup (A) intra-erythrocyte magnesium, (B) intra-erythrocyte zinc, and (C) intra-erythrocyte copper levels

No retinopathy was seen in the cases with T1DM. Fourteen of the cases (32.6%) had nephropathy at the microalbuminuric level. Table 2 shows the comparison between serum Mg and other blood parameters by group. No significant difference was found in the baseline eMg1, eZn1, eCu1, and uMg1 levels between the two groups (Figure 1, Table 2). When the ROC curve analysis was performed for eMg1, eZn1, eCu1, and uMg1, the area under curve was 0.613 [p=0.073, 95% confidence interval (CI): 0.470-0.756] for Mg, 0.526 (p=0.727, 95% CI: 0.383-0.668) for Zn, and 0.488 (p=0.869, 95% CI: 0.345-0.631) for Cu. Based on these values, the cut-off was estimated as 4.21 mg/dL for Mg and 845 µg/dL for Zn. When the normal and low levels were determined based on the cut-off values, the rate of low eMg1 level was higher in group 1 than in group 2. The rate of low eZn1 did not differ between the groups (Table 3).

Table 4 shows the eMg1, Zn, and Cu levels before and after iv MgSO4 infusion in group 1. These levels were significantly increased (p<0.001) after the iv Mg tolerance test. The Mg excretion ratio of 90.2±6.1% was measured from the urine collected after the iv MgSO4 infusion. In the study group, the overall ratio of Mg excretion was >50%.

No statistically significant difference was found in the eMg1, eZn1, eCu1, and uMg1 levels among patients based on their diabetic metabolic control state according to HbA1c levels (p>0.05), as shown in Table 5. Mg excretion ratios were similar in patients with well-controlled (89.3±6.9%) and poorly controlled (91.3±4.5%) diabetes (p=0.287). A positive correlation was found between HbA1c and urinary microalbumin/creatinine ratios (r=0.442, p=0.003). However, no statistically significant association was found between urinary microalbumin/creatinine ratios and eMg1 or uMg1 levels (r=0.068, p=0.663 and r=0.044, p=0.780, respectively).

## DISCUSSION

There is a growing interest for studies aiming to clarify the role of trace elements in the etiopathogenesis and complications of diabetes mellitus. The body reserve of trace elements is ideally measured at tissue level (16). Mg tolerance test is a reliable method that can well demonstrate the Mg level in tissue, but it requires short-term hospitalisation (17). In the present study, we found no statistically significant difference in the eMg1, eZn1, and eCu1 levels between patients and control subjects. Multiple factors may lead to Mg deficiency in T1DM patients. Possibly, the most important mechanism is the urinary Mg loss resulting from osmotic diuresis due to the hyperglycemia. Taurine deficiency, changes in vitamin D metabolism, intestinal absorption inadequacy, defects in glutathione metabolism are the other important factors (1,18,19). Recently, numerous studies have reported lower Mg levels in the plasma and tissue of diabetics (1,2,8). In our study, although serum Mg and eMg1 levels were similar in both groups, eMg1 tended to be lower in patients with T1DM than in the controls. Consistent with our study, Rohn et al (19) did not find any significant difference in the eMg1 levels between patients and controls, but they found a similar tendency for Mg deficiency in patients with T1DM. Sjögren et al (20) reported that eMg1 measurement was inadequate in evaluating the total body Mg reserve and that intra-leukocyte muscle Mg measurement could be a better method. Resnick et al (21) examined intracellular Mg deficiency among diabetics and found significantly decreased serum ionised Mg levels in these patients. No significant difference was found in the eMg1 level and urinary Mg excretion between the two groups in our study. This finding may be due to the sufficient dietary Mg intake of the patient group included in this study. In some studies in which normal trace element levels were found among diabetics, this finding was also associated with sufficient dietary Mg intake (22,23).

In some previous studies, Mg deficiency was implicated in the development of micro-and macro-vascular complications of diabetes (8,24,25). Hypomagnesemia is known to be associated with dyslipidemia, inflammatory load, and increased oxidative stress (26). The serum Mg level was found to be low in diabetic patients with concomitant hypertension, ischemic heart disease (27), and severe diabetic retinopathy (28). Mg deficiency accelerated atherosclerosis and vascular damage in experimental animals, and it has been reported that Mg replacement decreased the development of atherosclerotic lesions, thus reducing serum cholesterol and triglyceride levels (29,30). Atabek et al (8) showed that Mg deficiency was related to atherosclerotic changes independent of lipid levels in children with T1DM. We did not find a significant association between urinary microalbumin/creatinine ratios and eMg1 or urinary microalbumin/creatinine ratios and uMg1 levels.

In the study of Schnack et al (31), serum and eMg1 levels were inversely correlated with metabolic control among diabetics. Such a correlation was not found in our study, and there were also no significant differences in eMg1 levels between the patient and the control groups. We therefore suggest that the measurement of the intra-erythrocyte or the urinary Mg level may be insufficient for evaluating the total body Mg reserve in diabetic patients. The results of Sjögren et al (20) who also did not find any correlation between HbA1c and eMg1 or urinary Mg excretion, but found a significant correlation between muscle and the mononuclear intracellular Mg and HbA1c, support our suggestion.

In the present study, although the eMg1 level seems to be unaffected in diabetic patients, the mean urinary Mg retention of 90.2±6.1% and the significant increase in the eMg1, Zn and Cu levels following iv MgSO4 administration suggest an insufficient total body Mg reserve. Increased retention following the iv MgSO4 tolerance test is superior to other methods in demonstrating Mg deficiency (32,33). Similarly, Simşek et al (15) found >50% Mg retention in 43% of patients with T1DM. By contrast, some studies found that increased urinary Mg excretion was not related to serum Mg concentration (34).

Mg shows multiple effects by acting on bone mineral homeostasis, on stabilisation of the crystal structure as well as on calcium metabolism and ALP efficiency. ALP is known to express during the early stage of stiff bone tissue formation in bone and calcified cartilage. Although the effects of ALP on bone mineralisation are not fully understood, ALP is considered to enable the increase in inorganic phosphate (35). In our study, while the intracellular increase in trace elements after iv MgSO4 application indicated total body deficiency, a statistically significant increase in ALP supported the presence of such a deficiency in the diabetic group. ALP may increase to provide adequate functioning during Mg deficiency when the major portion of the Mg is present in the bone tissue. These results suggested that eMg1 level remained incapable to demonstrate the total body Mg concentration in diabetic patients.

In conclusion, eMg1 measurement cannot reveal Mg deficiency, but increased retention following iv Mg tolerance test indicates intracellular Mg deficiency in patients with T1DM. Moreover, if Mg deficiency is detected by the Mg retention test, which should be performed at least once a year, then Mg replacement therapy may help to provide glycemic control in poorly controlled T1DM patients.

## Ethics

Ethics Committee Approval: The study protocol was approved by the Ethics Committee of Osmangazi University Faculty of Medicine, Informed Consent: Informed consent was obtained from all included children and their parents.

Peer-review: External peer-reviewed.

## Figures and Tables

**Table 1:**
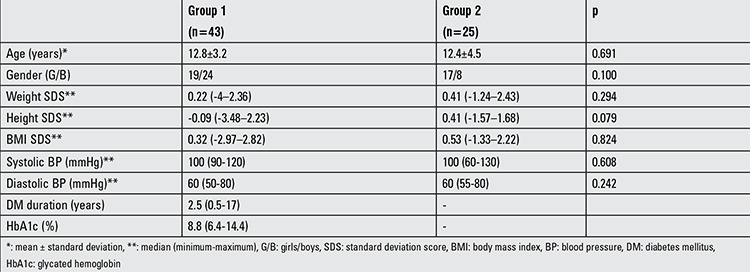
Demographic and anthropometric characteristics in the study and control groups

**Table 2:**
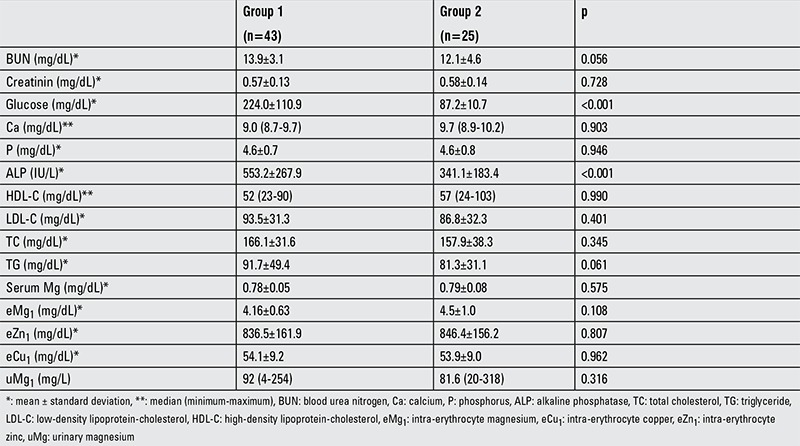
Comparison of biochemical parameters by group

**Table 3:**
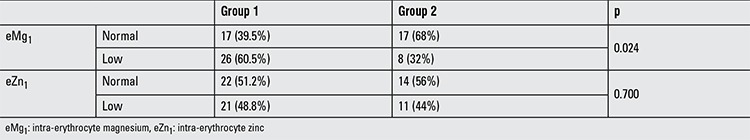
Numbers (ratios) of decreased intra-erythrocyte magnesium and intra-erythrocyte zinc levels by group

**Table 4:**
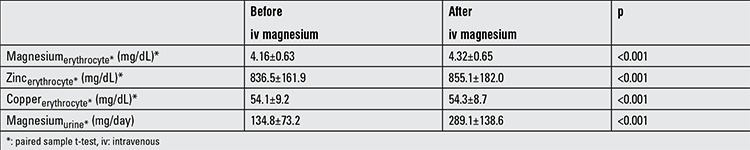
Intra-erythrocyte trace element levels before and after iv magnesium infusion in the patient group

**Table 5:**
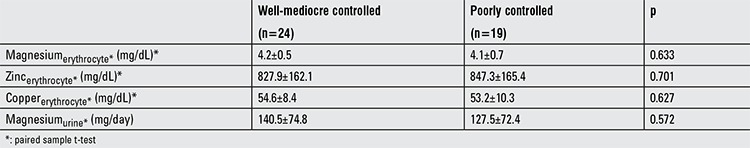
Differences in trace element levels by state of metabolic control
